# MetaGSCA: A tool for meta-analysis of gene set differential coexpression

**DOI:** 10.1371/journal.pcbi.1008976

**Published:** 2021-05-04

**Authors:** Yan Guo, Hui Yu, Haocan Song, Jiapeng He, Olufunmilola Oyebamiji, Huining Kang, Jie Ping, Scott Ness, Yu Shyr, Fei Ye

**Affiliations:** 1 Comprehensive Cancer Center, University of New Mexico, Albuquerque, New Mexico, United States of America; 2 Department of Biostatistics, Vanderbilt University Medical Center, Nashville, Tennessee, United States of America; 3 Division of Epidemiology, Department of Medicine, Vanderbilt University Medical Center, Nashville, Tennessee, United States of America; 4 Vanderbilt Center for Quantitative Sciences, Vanderbilt University Medical Center, Nashville, Tennessee, United States of America; Johns Hopkins University, UNITED STATES

## Abstract

Analyses of gene set differential coexpression may shed light on molecular mechanisms underlying phenotypes and diseases. However, differential coexpression analyses of conceptually similar individual studies are often inconsistent and underpowered to provide definitive results. Researchers can greatly benefit from an open-source application facilitating the aggregation of evidence of differential coexpression across studies and the estimation of more robust common effects. We developed Meta Gene Set Coexpression Analysis (MetaGSCA), an analytical tool to systematically assess differential coexpression of an *a priori* defined gene set by aggregating evidence across studies to provide a definitive result. In the kernel, a nonparametric approach that accounts for the gene-gene correlation structure is used to test whether the gene set is differentially coexpressed between two comparative conditions, from which a permutation test *p*-statistic is computed for each individual study. A meta-analysis is then performed to combine individual study results with one of two options: a random-intercept logistic regression model or the inverse variance method. We demonstrated MetaGSCA in case studies investigating two human diseases and identified pathways highly relevant to each disease across studies. We further applied MetaGSCA in a pan-cancer analysis with hundreds of major cellular pathways in 11 cancer types. The results indicated that a majority of the pathways identified were dysregulated in the pan-cancer scenario, many of which have been previously reported in the cancer literature. Our analysis with randomly generated gene sets showed excellent specificity, indicating that the significant pathways/gene sets identified by MetaGSCA are unlikely false positives. MetaGSCA is a user-friendly tool implemented in both forms of a Web-based application and an R package “MetaGSCA”. It enables comprehensive meta-analyses of gene set differential coexpression data, with an optional module of *post hoc* pathway crosstalk network analysis to identify and visualize pathways having similar coexpression profiles.

## Author summary

Analyses of gene set differential coexpression often shed light on molecular mechanisms underlying phenotypes and diseases. However, results from conceptually similar individual studies are often inconsistent and underpowered to reach definitive conclusions. We provide an open-source application facilitating the aggregation of evidence of differential coexpression across studies and the estimation of more robust common effects, with an optional module of *post hoc* pathway crosstalk network analysis to identify and visualize pathways having similar coexpression profiles. We established the usefulness of MetaGSCA via case studies of chronic kidney disease and non-small cell lung cancer, and applied it to a pan-cancer analysis of 11 cancer types. We further demonstrated the tool with 100 randomly generated gene sets and showed excellent specificity, indicating low false positive rates.

## Introduction

Compared to conventional differential expression approaches where genes are evaluated individually assuming gene independence, differential coexpression analysis interrogates gene-gene co-transcription relations and represents a complementary perspective into diseased transcriptomes. Although transcriptome data are typically analyzed to find differentially regulated individual genes, an alternative analysis strategy exists that aims to identify sets of potentially correlated genes that, together, explain a significant proportion of phenotypic variance [1–3]. The key idea is to quantify the strength of the similarity or dependency calculated from pairwise gene expression data using measures such as Pearson’s or Spearman’s correlation. A pioneering approach was proposed in 2005 [4] and later developed into the widely adopted software WGCNA [5]. Many studies used WGCNA to identify or verify differentially coexpressed gene sets. Other tools, such as CoXpress [6], GSCA [7], and GSNCA [8], were developed to identify extreme differential coexpressions. Unlike the purely data-driven tool CoXpress aiming to identify *a posteriori* gene sets, GSCA and GSNCA incorporate gene function knowledge to assess predefined functional groups, such as Gene Ontology terms or cellular pathways. While differential coexpression analyses have become increasingly popular for their potential of uncovering dysregulation mechanisms underlying human diseases, there are often barriers between informatics and biological researchers, which need to be bridged to translate omics data into valuable biological or medical discoveries. A key benefit of meta-analysis is the aggregation of information leading to a higher statistical power and a more robust point estimate than is possible from individual studies. Here, we propose a tool for aggregating data across studies to empower differential coexpression analysis in various contexts and facilitate meta-analysis of complex large gene expression datasets.

To reach these goals, we developed MetaGSCA (“Meta Gene Set Coexpression Analysis”) to systematically assess the differential coexpression pattern of an *a priori* gene set across studies. Within each study (or dataset), a kernel algorithm measures the strength of differential coexpression of the gene set between two conditions. MetaGSCA wraps a meta-analysis framework around the kernel algorithm to estimate an overall effect over individual studies and constructs nonparametric confidence intervals via bootstrapping to provide a measure of uncertainty in the point estimate. MetaGSCA encloses 379 cellular pathways (S1 Table) and provides a *post hoc* pathway crosstalk analysis module to identify and graphically delineate pathways having similar coexpression profiles across studies.

## Results

### MetaGSCA design and implementation

**Fig 1:**
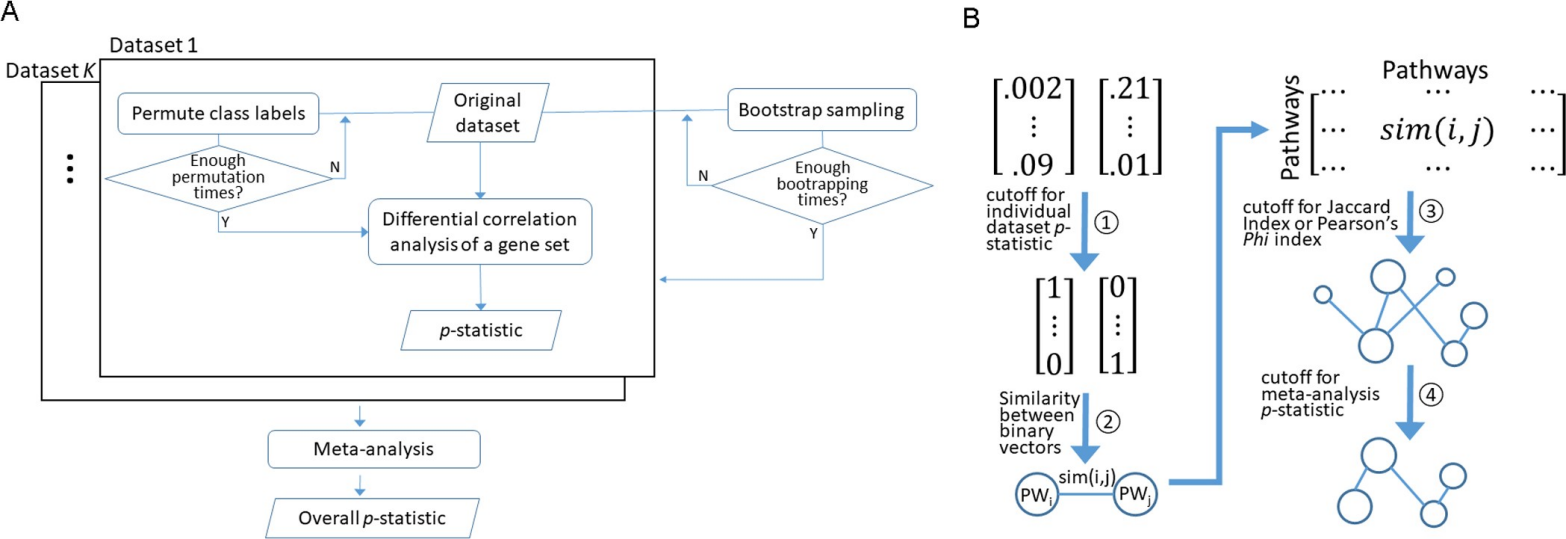
MetaGSCA schema. A) Meta-analysis of gene set differential coexpression. The kernel algorithm is based on the GSNCA method and yields a *p*-statistic for each individual study or dataset. MetaGSCA wraps a meta-analysis framework around the kernel algorithm to estimate an overall effect over individual studies and constructs nonparametric confidence intervals via bootstrapping. B) Pathway crosstalk analysis is performed as an optional step following the meta-analysis. In the schematic network, node size is proportional to the overall *p*-statistic estimated in the meta-analysis; a threshold of significance is applied to filter non-significant pathways. PW_i_: the *i*^th^ pathway; PW_j_: the *j*^th^ pathway.

MetaGSCA consists of three main modules: differential coexpression analysis for individual datasets, meta-analysis over multiple datasets, and pathway crosstalk network analysis. The overall structure of MetaGSCA is illustrated in Fig 1. MetaGSCA was developed primarily in R. The companion web application was designed with a combination of PHP, JavaScript, and HTML. We conducted a runtime analysis on MetaGSCA with four parameters: number of genes, number of datasets, number of permutations, and number of bootstrap repetitions. One parameter was tested at a time and the rest of the parameter vector was kept constant at default values. The test machine used was a Windows 10 with Intel Xeon CPU E5-1650 at 3.6 GHz and 32 GB RAM. The results (S1 Fig) show that the run time of MetaGSCA scales with all four parameters. Actual runtime may vary depending on the CPU speed.

### Case studies and specificity analysis

**Fig 2:**
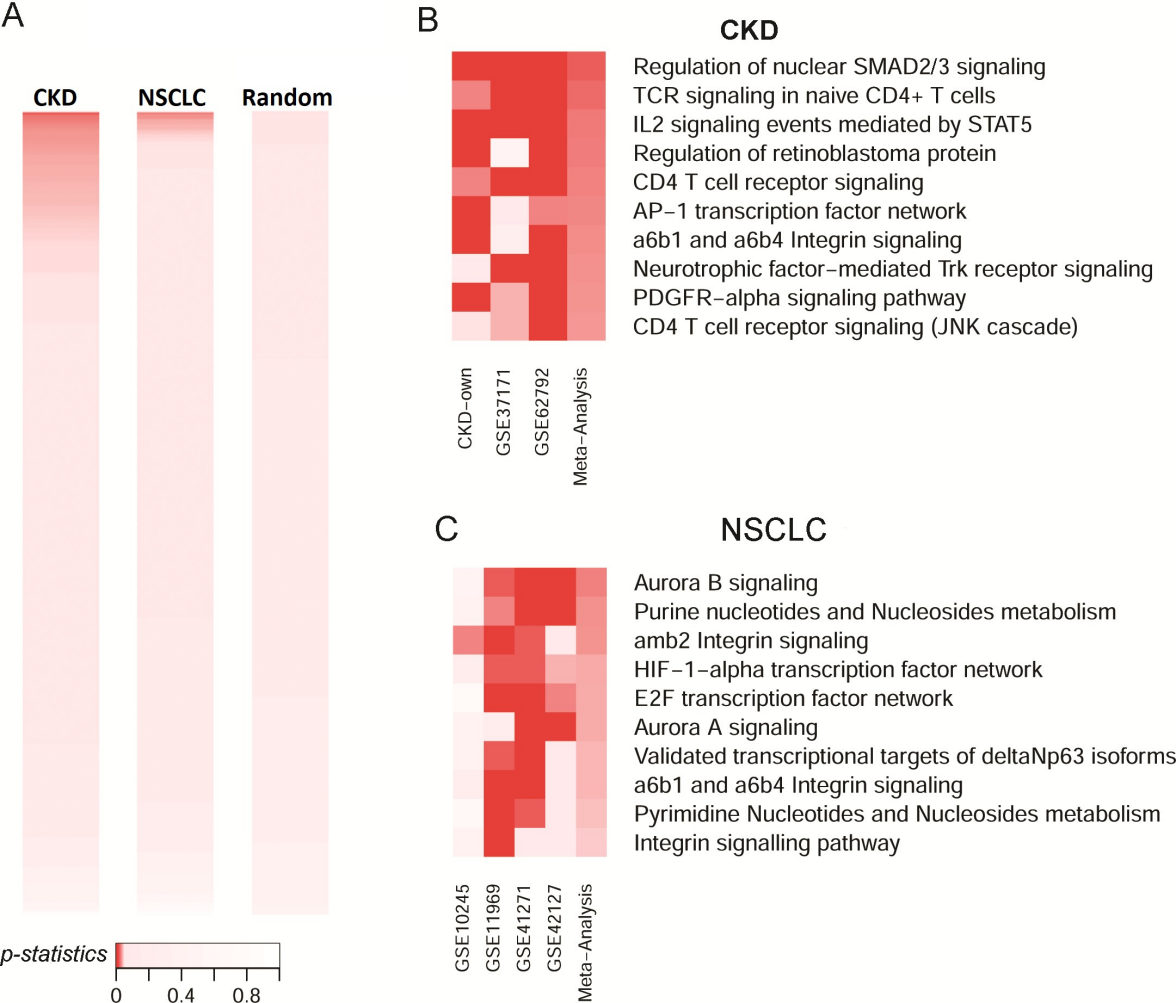
Case studies and specificity analysis. A) Meta-analysis *p*-statistics from the CKD and NSCLC case studies as well as 100 random gene sets. B) Top 10 significant pathways identified in CKD. C) Top 10 significant pathways identified in NSCLC. For both case studies, *p*-statistics from all individual datasets and the meta-analysis are displayed in a heatmap.

To demonstrate MetaGSCA’s ability in the identification of known disease-associated pathways, we conducted two case studies, one in chronic kidney disease (CKD) and one in non-small cell lung cancer (NSCLC). The results of the CKD and NSCLC case studies are visualized in side-by-side color bars (significance threshold *p*<0.05) (Fig 2A). The CKD meta-analysis identified 77 dysregulated pathways, and the NSCLC meta-analysis identified 16.

In our previous work with the three datasets in the CKD case study, we attempted to integrate the GSNCA results from individual datasets with ad hoc criteria [9]. Among the top 10 CKD-relevant pathways prioritized by MetaGSCA, 7 were identified in our previous study. The remaining 3 (*Regulation of retinoblastoma protein*, *PDGFR-alpha signaling pathway*, *and CD4 T cell receptor signaling*) were missed because they had a *p*-value slightly higher than the significance threshold in one of the individual datasets. In the current MetaGSCA, the estimated overall effects showed that these pathways are significant across studies and worth further investigation. Notably, *Regulation of nuclear SMAD2/3 signaling*, a pathway with direct implication in chronic kidney disease [10,11], was prioritized at the very top of the meta-analysis significant results (Fig 2B). This suggests that MetaGSCA provides a more streamlined and reliable approach to prioritize pathways that showed differential coexpression across studies/datasets.

In the NSCLC case study of four datasets, among the top 10 pathways identified by MetaGSCA (*p*-statistic<0.05), three are related to integrins, namely *amb2 Integrin signaling*, *a6b1 and a6b4 Integrin signaling*, and *Integrin signaling pathway* (Fig 2C). Integrins are important players in cell junctions, and cell junctions have been proved to be critical in differentiating lung squamous cell carcinoma and lung adenocarcinoma [12]. The two Aurora signaling pathways (*Aurora A signaling* and *Aurora B signaling*) are also related to the survival and drug resistance of NSCLC [13].

To evaluate the specificity of MetaGSCA, we tested it with 100 random gene sets generated from The Cancer Genome Atlas (TCGA) data, each consisting of 10 to 20 randomly selected genes. The study design of both paired and unpaired analyses is the same as the pan-cancer application described in the next section. Among the 100 random gene sets analyzed by MetaGSCA, none were found with significant coexpression in the unpaired analysis; and only 4 and 3 were found significant in the paired analysis by the generalized linear mixed model (GLMM) and inverse variance method, respectively. These results showed a very high specificity (96–100%), suggesting that the significant differential coexpression patterns identified in the case studies are most likely true positives.

### A pan-cancer application

**Fig 3:**
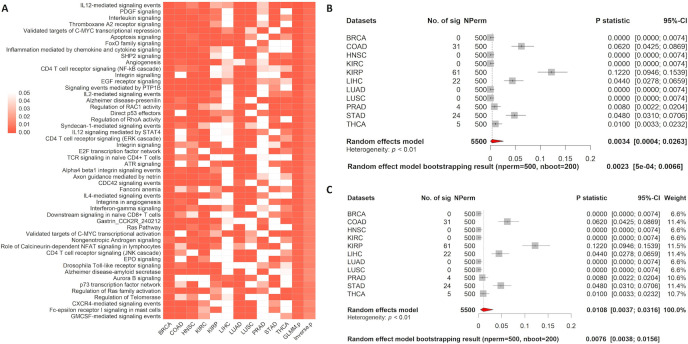
Pan-cancer MetaGSCA analysis in 11 cancer types, paired. A) The heatmap displays the top 50 pathways that MetaGSCA identified. The first 11 columns are the bootstrap *p*-statistic for each cancer type. Columns 12 and 13 are the meta-analysis *p*-statistics for the GLMM and the inverse variance method, respectively. B) Forest plot for the meta-analysis results of the *valine*, *leucine*, *and isoleucine degradation pathway* with the GLMM option. C) Forest plot for the meta-analysis results of the *valine*, *leucine*, *and isoleucine degradation pathway* based on the inverse variance method. In the forest plot, the dotted line denotes the meta *p*-statistic. Individual studies having stronger differential coexpression than the overall effect are on the left of the dotted line.

To further evaluate MetaGSCA’s ability in analyzing both paired and unpaired data, we conducted a pan-cancer analysis using TCGA data. A detailed description of paired and unpaired data is available in the Materials and methods section. In the unpaired analysis, 268 and 245 pathways were found to be significantly dysregulated by the GLMM and inverse variance methods, respectively. The results for the top 50 pathways are presented in Fig 3A. The top pathway is the *IL12 signaling mediated by STAT4 pathway*. IL12 is the major instructive cytokine signal-boosting the ability of CD8(+) T cells to express CD40L and has been used extensively in T-cell immunotherapy to treat cancers [14]. The forest plots of the IL12 signaling mediated by STAT4 show that the pathway is differentially coexpressed between tumor and normal in 9 out of the 11 cancer types (*p*-statistics<0.05). The meta-analysis indicates strong overall pathway coexpression across cancer types (*p*-statistic: 0.002 and 0.008 respectively, by the GLMM and inverse variancef methods) (Fig 3B and 3C). These results suggest that the *IL12 signaling mediated by STAT4 pathway*’s coexpression is frequently altered during tumorigenesis.

**Fig 4:**
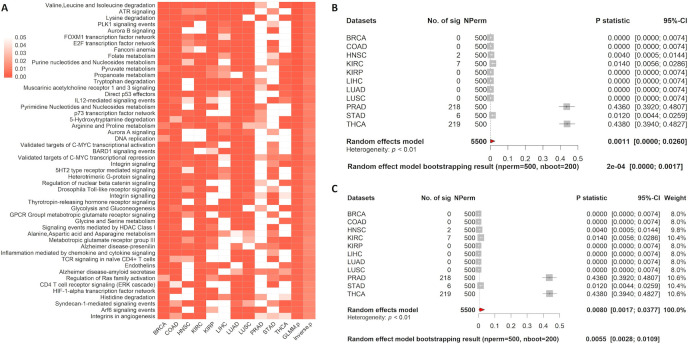
Pan-cancer MetaGSCA analysis in 11 cancer types, unpaired. A) The heatmap displays the top 50 pathways that MetaGSCA identified. The first 11 columns display the bootstrap *p*-statistic for each cancer type. Columns 12 and 13 are the meta-analysis *p*-statistic for the GLMM and the inverse variance method, respectively. B) Forest plot for meta-analysis results of the *IL12 signaling mediated by STAT4 pathway* with the GLMM option. C) Forest plot for meta-analysis results of the *IL12 signaling mediated by STAT4 pathway* based on the inverse variance method. In the forest plot, the dotted line denotes the meta *p*-statistic. Individual studies having stronger differential coexpression than the overall effect are left of the dotted line.

In the paired analysis, 298 and 255 pathways were found to be significantly dysregulated by the GLMM and inverse variance methods, respectively. The results for the top 50 pathways are summarized in Fig 4A. The top dysregulated pathway is the *valine*, *leucine*, *and isoleucine degradation pathway* for both methods. Valine, leucine, and isoleucine are essential amino acids used in the biosynthesis of proteins; all three have been linked to cancer. For example, valine can be reprogramed by targeting HIBCH to treat colorectal cancer [15], leucine deprivation has been shown to inhibit proliferation and induces apoptosis in breast cancer cells [16], and isoleucine has been shown to prevent liver metastases [17]. The forest plots of the valine, leucine, and isoleucine degradation pathway show that the pathway is differentially coexpressed between tumor and normal in 9 out of the 11 cancer types (*p*-statistics<0.05). The meta-analysis indicates a strong overall pathway coexpression across cancer types (*p*-statistic: 0.0002 and 0.0055, respectively, by the GLMM and inverse variance methods) (Fig 4B and 4C). These results suggest that the *valine*, *leucine*, *and isoleucine degradation pathway* is frequently altered during tumorigenesis in many types of cancers.

**Fig 5:**
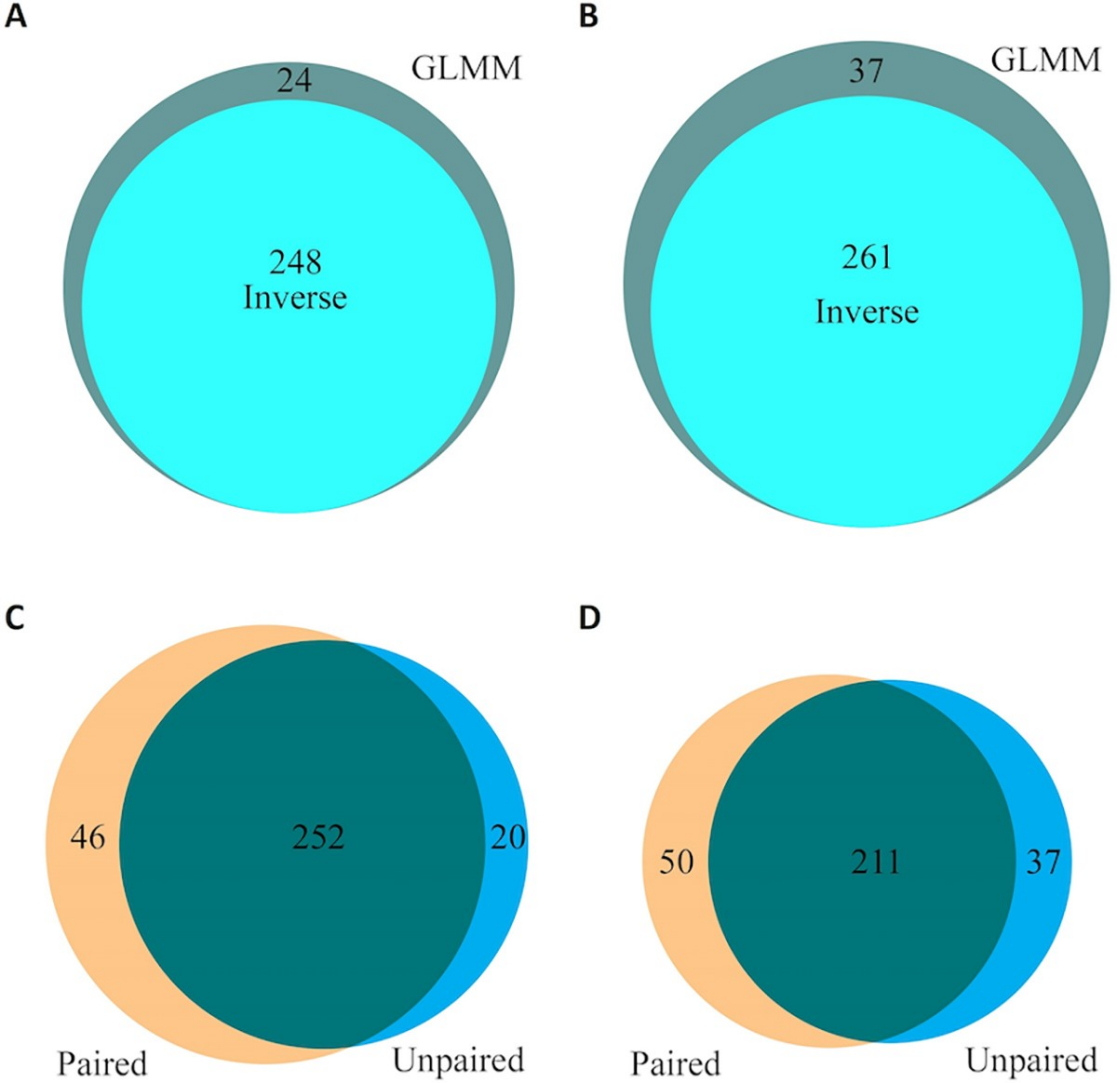
Consistency and comparison of pathways identified by GLMM and inverse variance in the paired and the unpaired analyses. A) Consistency between GLMM and inverse variance in the paired analysis. B) Consistency between GLMM and inverse variance in the unpaired analysis. C) Comparison between the paired and unpaired analyses by the GLMM method. D) Comparison between the paired and unpaired analyses by the inverse variance method.

We further summarized the analysis results between the GLMM and the inverse variance method with the default logit transformation. Regardless of pairing, both models generated very similar results, though the GLMM seemed to be slightly more sensitive as it found all significant pathways identified by the inverse variance method (Fig 5A and 5B). Both models identified more significant pathways in the paired analysis than the unpaired analysis (Fig 5C and 5D). Since statistical power depends on sample size as well as correlation structure in the data, results based on statistical significance are not directly comparable between the unpaired and paired analyses. Though the majority of the significant pathways identified by paired and unpaired analyses were common, each identified many pathways specific to the study design.

### Pathway crosstalk analysis

**Fig 6:**
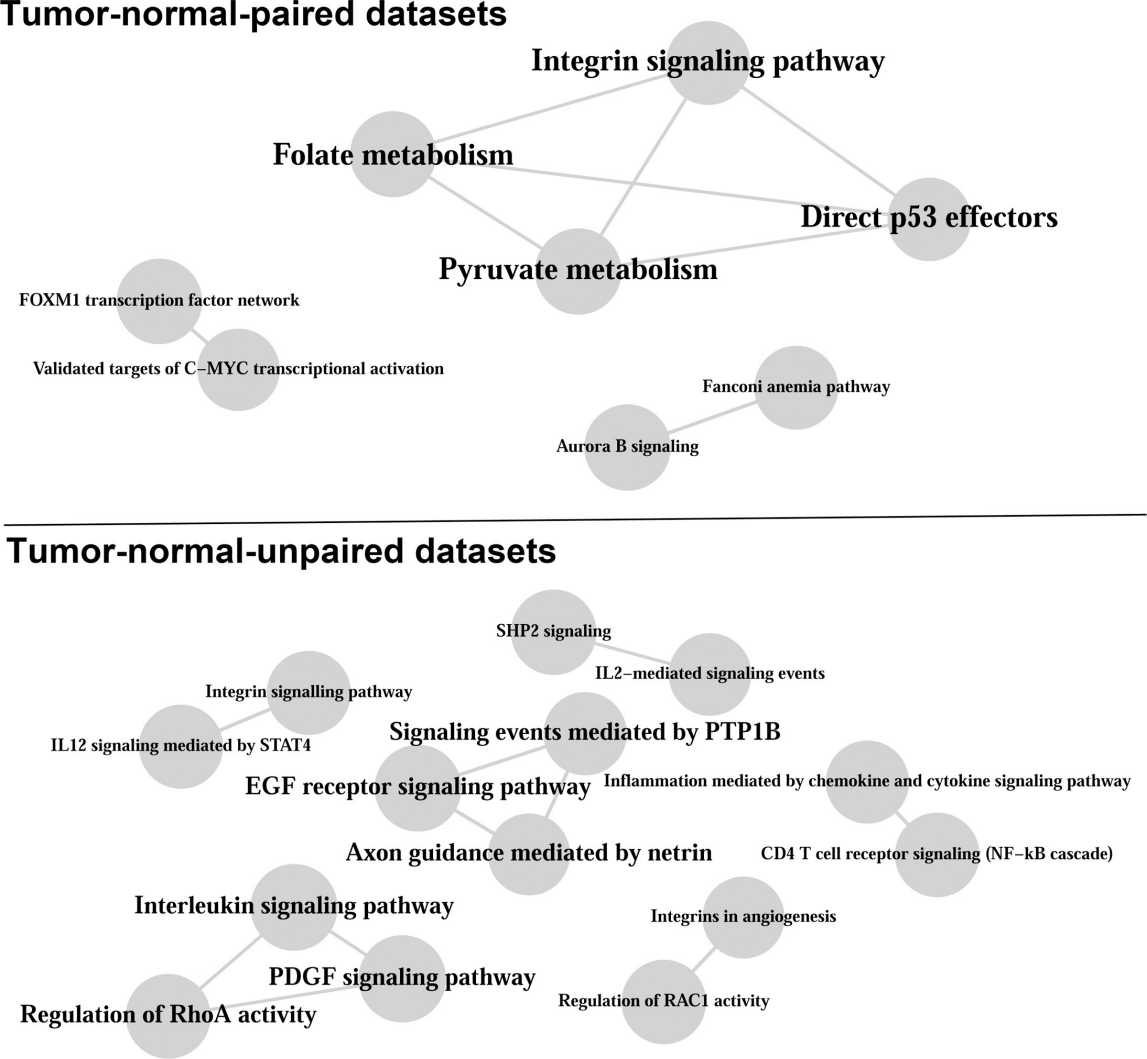
Pathway crosstalk network inferred from the meta-analysis of gene set differential coexpression across transcriptome datasets of 11 cancers. Significance thresholds of individual dataset *p*-statistic, meta-analysis *p*-statistic, and *p*-value of Pearson’s phi were all set at 0.01.

MetaGSCA provides an optional module to facilitate pathway crosstalk analysis. This function was demonstrated using the results from the TCGA pan-cancer analysis (Fig 6). Pathway crosstalk networks were constructed separately for the paired and unpaired analyses and included only pathways found significant in the meta-analysis (*p*-statistic<0.01). Pathway similarity was quantified using Pearson’s phi to describe connectivity between pathway pairs. Two pathways were connected if Pearson’s phi *p*-value<0.01. The crosstalk network analysis returned 8 connections with 8 pathways from the paired data and 10 connections with 14 pathways from the unpaired data. These two networks shared only one pathway vertex (the *Integrin signaling pathway*) and therefore had no common connections. As an alternative approach, we performed the pathway crosstalk analysis using the Jaccard similarity coefficient, which generated a bigger network that contains the network created by Pearson’s phi. Overall, our pathway crosstalk analysis protocol showed satisfactory robustness against varying parameters and variations in the data. In the current analysis, we chose a relatively stringent cutoff (*p*-statistic<0.01) to demonstrate this feature of MetaGSCA. A relaxed threshold would lower the sensitivity in detecting coexpressed pathways, therefore include more pathways in the crosstalk analysis. On the other hand, a more stringent threshold may be used to minimize the rate of false positives. Of course, the results will also depend on the sample size and study design of individual studies in the meta-analysis.

## Discussion

Differential coexpression network analysis has been used increasingly to explore the systemic functionality of genes. As the number of available genomic datasets grows exponentially, there is also a rising demand for aggregating genomic data from multiple datasets or studies to provide more robust statistical estimates with a measure of uncertainty for gene set differential coexpression analysis. In this paper, we combined the strengths of differential coexpression network analysis and meta-analysis to develop MetaGSCA, which allows for a systematic meta-evaluation of the differential coexpression of a gene set between two conditions. Testing of MetaGSCA on tumor RNAseq expression data from 11 cancer types demonstrated high sensitivity, and negative trials using randomly generated gene sets demonstrated excellent specificity.

A weakness of meta-analysis is that sources of bias are not controlled by the method but depend on the design and availability of covariate data in the original studies. In the paired analysis, since each patient served as their own control, no confounding issues would arise from factors such as age and sex. In the unpaired analysis, the overall effect in the meta-analysis may be computed from effect estimates adjusted for covariates in individual studies. In the meta-analysis, two different approaches are available for estimating the pooled overall effect provided by the R function “*metaprop*”: the inverse variance method and the GLMM with a random intercept logistic regression model. Recommendations in the literature regarding the choice of transformation are summarized in the R documentation [18]. Overall, the GLMM with logit transformation appears to be more popular than other options, although individual study weights are not available by the method as it uses a random intercept.

There is a plethora of cancer-related pathways identified in the pan-cancer analysis. For example, the *angiogenesis pathway* was identified as significant for 10 out of 11 cancer types for both the GLMM and inverse variance methods. Angiogenesis is the physiological process through which new blood vessels form from pre-existing vessels, and is important because cancer cell proliferation and metastatic spread require sufficient oxygen and nutrients delivered through the vascular system [19]. Multiple angiogenesis inhibitors have been developed as cancer treatments [20]. Other noteworthy pathways are the *thromboxane A2 receptor signaling pathway* and the *TCR-signaling pathway*. *Thromboxane A2 receptor signaling pathway* has been hailed as an emerging paradigm in cancer progression and metastasis [21]. The *TCR-signaling pathway* is a T-cell immunotherapy related pathway which has direct implications in chimeric antigen receptor t-cell (CAR-T) cancer treatment [22]. A potential confounding effect to differential coexpression analysis can arise from differential expression enriched with tissue-specific genes [23]. The authors found that often differential coexpressions detected are not ‘pure’ differential coexpressions—defined as links between genes expressed in all tissues. Our pan-cancer analysis using the TCGA gene expression data provides a practical example for identifying pathways that are common across tissues for further investigations. By identifying which pathways are likely to be present in many tissues, it also helps us to identify which pathways are enriched for tissue-specific genes. Analogous to this, cell-type deconvolution analysis of (bulk) gene expression data of tissues made up a mixture of cell types is considered important because of the confounding effect of cell type composition differences in gene expression samples. The MetaGSCA package can be potentially used to identify gene signatures that are enriched in particular cell types across data sets and gene signatures that are present in many cell types.

Currently, MetaGSCA implements GSNCA [8] as the core individual-dataset coexpression algorithm that outputs the permutation-based *p*-statistic. The meta-analysis framework of MetaGSCA, however, is compatible with any algorithm designed for estimating gene set differential coexpression in a single dataset. Future work will focus on the investigation of alternative test statistics and algorithms to provide more methodological flexibility for users.

## Materials and methods

### Development

MetaGSCA is developed in the R environment as a tool to empower meta-analysis of gene set differential coexpression (Fig 1). Theoretically, MetaGSCA can be built upon any common gene set differential coexpression algorithm. The current software adopts the GSNCA algorithm, a nonparametric test that assesses multivariate changes in the gene coexpression network of a gene set between two conditions. A gene set is a group of functionally coherent genes, which can be a pathway of interest, a signature reported in the literature, or a custom set of genes derived from preliminary analysis on the basis of biological relevance and/or statistical significance.

### Meta-analysis of gene set differential coexpression

An abstract depiction of the meta-analysis wrapping around gene set coexpression is given in Fig 1A. Given one individual dataset involving two comparative conditions, in order to estimate the differential coexpression of a gene set between the two conditions, a conceptual full-connection gene coexpression network is created under each condition, where the nodes represent the genes, and the edges connect genes. The strength of the connection is given by the edge weights. Suppose we have *K* individual studies containing a gene set with expression profiles of *q* genes in two comparative conditions. Focusing on one of the *K* studies, for each condition *l* (*l* = 1,2), let *R*_*l*_ with elements *r*_*ij*_ denote a *q*×*q* gene correlation matrix for each condition and *N*_*l*_ denote the coexpression network connecting the two conditions, with *q* nodes (genes) and *q*(1−*q*)/2 edges. The weight of an edge between two nodes (genes) *i* and *j* is given by 1−|*r*_*ij*_| and can be considered to be the correlation distance, where the *r*_*ij*_′s are pairwise correlations.

In GSNCA, genes are assigned with weight factors that are computed proportionally to cross-correlation (i.e., gene-gene correlations represented by edge weights) [8]. Denote the weight of a gene as *w*_*i*_: *w*_*i*_ = ∑_*j*≠*i*_*w*_*j*_*r*_*ij*_, 1≤*i*≤*q*. In a nutshell, an eigenvector is derived from the full-connection correlation matrix, whose elements are assigned as weights of the gene vertices in the network. Each gene, therefore, obtains a weight value that characterizes its cross-correlation strength with all other genes in the coexpression network *N*_*l*_. Let the weight vector ***w***_***l***_ be the first eigenvector (the eigenvector with the largest eigenvalue) of the *q*×*q* gene correlation matrix *R*_*l*_. Thus, two weight vectors, ***w***_**1**_ and ***w***_**2**_, can be constructed for condition 1 and condition 2, respectively. The test statistic capturing differences in gene set coexpression between the two conditions is defined as the *L*_1_ norm between the two scaled weight vectors: $d\;=\;\sum_{i\;=\;1}^{q}|w_{1}\left[i\right]-w_{2}[i]|$, where each weight vector is multiplied by its norm. Because the *d* statistic does not follow a known distribution, a *p*-value cannot be directly computed from the *d* statistic. Permutations are generated to construct an exact test for which the distribution of the *d* statistic under the null hypothesis is obtained by randomly labeling samples across conditions (e.g., cancer vs. normal). The *p*-value of the permutation test is calculated by the proportion of the *d* test statistics falling into the rejection region in the permutations; that is, the proportion of *d* test statistic values of the permutations that are at least as extreme as the test statistic calculated from the original data. Let *nperm* be the number of permutations, *d*^*obs*^ be the observed value of the test statistic and $d_{i}^{perm}$ be the permutation test statistic, such that the permutation $p\;=\;\frac{\sum_{i\;=\;1}^{nperm}I\left(d_{i}^{perm}\geq d^{obs}\right)+1}{nperm+1}$. A low value suggests a rejection of the null hypothesis that the two corresponding weight vectors are equal. To avoid confusion, we refer to the proportion of *d* test statistics falling into the rejection region as the *p*-statistic in the rest of the paper, which will be used in the meta-analysis as the summary statistic to describe the overall effect size.

The *p*-statistic provides a point estimate of the strength of differential coexpression. The precision of its estimate can be addressed with a confidence interval [24,25]. We use bootstrapping [26] to provide an nonparametric estimate and to supplement the point estimate with a confidence interval. In this work, we use the bootstrap method to repeatedly draw random samples from the original dataset with replacement. With each bootstrap sample, we calculate a permutation *p*-statistic. Suppose the same bootstrap procedure is repeated *nboot* times, we then create a list of *p*-statistics,${\;p}_{boot}\;=\;p_{boot}^{\left(1\right)},\;p_{boot}^{\left(2\right)}\ldots \;p_{boot}^{\left(nboot\right)}$, from which the mean, median, standard error, and confidence interval can be derived. Without making a normality assumption about the sampling distribution, the 2.5^th^ and 97.5^th^ percentiles of the bootstrap sampling distribution approximate the 95% confidence interval of the estimated permutation test *p*-statistic under the null in *nboot* repetitions. While the theory of permutation tests is based on the idea of looking at every possible permutation of the test statistic, in practice, it is often not feasible to make the permutations exhaustive to compute an exact *p*-value. And because of that, approximate permutation tests are often conducted with a large number of resamples. The bootstrap interval for the *p*-statistic, therefore, provides a measure of uncertainty about the permutation test. If the confidence interval contains the significance level threshold based on Type I error, it suggests the number of permutations (*nperm*) needs to be larger. In other words, if the number of permutations is sufficiently large, the confidence interval will be narrow enough, and a decision to reject the null hypothesis or not to reject it can be confidently made. The bound of the confidence interval that is closer to the significance level threshold can be used as a conservative estimate.

The main aims of meta-analyses are to obtain an overall estimate of an effect and determine whether the effect exists across studies or datasets. To summarize the strength of gene set differential co-expression over multiple studies, we propose a meta-analysis approach. The R packages *meta* [27] and *metafor* [18] allow users to choose between the fixed- and random-effects models for a meta-analysis. If we choose the fixed-effects model, we assume that the parameter of interest is identical across studies and any difference between the observed *p*-statistics is only due to sampling error; if we choose the random-effects model, we assume that the observed difference between the proportions cannot be entirely attributed to sampling error and may be caused by other factors such as differences in study population, study designs, etc. Cochran’s Q test (Cochran, 1954) of heterogeneity can be performed in the meta-analysis to examine the finding’s consistency across studies. If heterogeneity is a concern, the random-effects model is recommended. In this case, each study provides a *p*-statistic, and the overall estimate describes their mean across studies. The variance parameter describes the heterogeneity among the studies (when the variance is zero, this is equivalent to the fixed-effects model). Sources of variability can be divided into within-study variance *ε*^2^ and between-study variance *τ*^2^. Under the fixed-effects model, the observed effect size is measured by ${\hat{ES}}_{k}\;=\;logit{\left(\hat{p_{1}}\right)}_{k}-logit{\left(\hat{p_{2}}\right)}_{k},\;k\;=\;1,2,\ldots ,\;K.$ The only source of uncertainty is the within-study variance $\varepsilon_{k}^{2}$, thus ${\hat{ES}}_{k}\;=\;{ES}_{k}+\varepsilon_{k}^{2},\;k\;=\;1,2,\ldots ,\;K$. Under the random-effects model, there is the between-study variance $\tau_{k}^{2}$ in addition to the same within-study variance, thus ${\hat{ES}}_{k}\;=\;{ES}_{k}+\varepsilon_{k}^{2}+\tau_{k}^{2},\;k\;=\;1,2,\ldots ,\;K$. The overall *p*-statistic can be estimated using two approaches. (1) The inverse variance method uses the transformed proportions and corresponding standard errors. In the R function “*metapro”*, options available are log transformation, Freeman-Tukey double arcsine transformation, arcsine transformation, and untransformed. (2) The GLMM with a random intercept logistic regression model that implicitly uses the logit transformation. With the *logit* transformation, the binary outcome ${I(d}_{i}^{perm}\geq d)$ is transformed and regressed on the study variable (i.e., cancer type). The *logit* function maps the probabilities of the binary outcome to the full range of real numbers (-∞, +∞): $\mathrm{logit}\left(p\right)\;=\ln\left(\frac{p}{1-p}\right),\;0\leq p\leq 1$. Confidence intervals estimated from both the model and the bootstrap distribution are provided.

### Crosstalk network analysis of pathway coexpression

Research interest in delineating pathway crosstalk networks dates back to as early as 2008 [28]. The evolving methodology mostly relies on gene-level connections between pathways, such as protein-protein interactions or gene coexpression data [29,30]. Here, we set out to delineate a pathway crosstalk network based on the meta-analysis result outputted from the previous steps of MetaGSCA. The resultant network reflects the similarity of analysis results between a pair of pathways over a spectrum of analogous or related datasets. Two pathways having similar profiles of gene set coexpression across studies are connected in the network.

In MetaGSCA, we provide a pathway crosstalk network module, which is relatively independent of the main components of MetaGSCA and allow users to execute it optionally. This module relies on the results of differential coexpression meta-analyses on many pathways (say, >50) and consists of the following empirical steps (Fig 1B): 1) convert the matrix of *p*-statistics of all pathways into a binary data matrix, with 1’s indicating the presence of differential coexpression, and 0’s the absence; 2) quantify pairwise pathway similarity by one of the two alternative measures—the asymmetric binary similarity that is equivalent to the Jaccard similarity coefficient and the Pearson’s phi index (with associated *p*-value); 3) apply a threshold on the pathway similarity; 4) reduce the pathway network by keeping connections incident to pathways that have a significant *p*-statistic. This pathway crosstalk analysis protocol uses three threshold parameters, separately applied to the *p*-statistics of individual datasets, the overall meta-analysis *p*-statistic, and the similarity measure between two pathways. The R package *iGraph* [31] is used to render the visual network layout.

### Pathway and gene expression data

Pathways were obtained from three primary pathway sources: PID [32], PANTHER [33], and INOH [34]. We enclosed a total of 379 cellular pathways as reference gene sets in MetaGSCA (S1 Table). The gene expression datasets involved in the case studies and the pan-cancer application are described in the next few sections and summarized in S2 Table.

### Case studies using MetaGSCA

We demonstrated MetaGSCA in two case studies of human diseases. The CKD study includes three independent gene expression datasets: one from our group [9] and two (GSE62792 [35] and GSE37171 [36]) from the Gene Expression Omnibus. The NSCLC study includes four independent gene expression datasets (GSE10245, GSE11969, GSE41271, and GSE42127), which were curated and processed in our previous work [37].

### Pan-cancer application using MetaGSCA

Additionally, we applied MetaGSCA to conduct a pan-cancer analysis. Gene expression data were downloaded from the TCGA Data Portal. In ~10% of TCGA samples, molecular profiling data of normal tissue adjacent to the tumor (NAT) were also generated. These NAT samples present a unique intermediate state between healthy and tumor and are commonly used as controls in cancer studies [38]. Therefore, we conducted the pan-cancer analysis parallel with the tumor-NAT pairs and all available tumor samples and NAT samples. We considered only cancer types having at least 30 tumor-NAT pairs, limiting to 11 different cancer types that altogether involved 4,617 tumor samples and 602 NAT samples. Pathways were required to contain at least ten genes to be included in this pan-cancer analysis.

Without making assumptions about homogeneity, we used a random-effects model. The calculation of the *d* statistic takes the following steps in both paired and unpaired analyses. (1) Patient samples were separated into two groups by condition (e.g., cancer vs. normal). The two groups can be of different sizes in the case of unpaired analysis. (2) Within each group (cancer or normal), a gene-gene correlation matrix *R*_*l*_ was constructed. The elements *r*_*ij*_ of the correlation matrix represents the strength of correlation between gene *i* and gene *j*. The (first) eigenvector was subsequently derived from the correlation matrix *R*_*l*_. (3) The test statistic d was then calculated as the L2 norm between the two groups (i.e., the distance between the eigenvectors).

### Specificity analysis

We generated 100 random gene sets to conduct the specificity analysis, each consists of 10–20 genes randomly selected from the TCGA gene expression datasets (therefore, no coexpression patterns were expected). MetaGSCA was applied to these 100 random gene sets following the same steps as in the pan-cancer application.

## Supporting information

S1 Fig MetaGSCA run time analysis. (TIFF)

S1 Table Original source of reference pathways enclosed in MetaGSCA. (DOCX)

S2 Table Summary of the expression datasets entailed in the two case studies and the pan-cancer application. (DOCX)
