# Supplementary figures and images for: MetaGSCA: A tool for meta-analysis of gene set differential coexpression
Source: PLoS Comput Biol. 2021 May 4;17(5):e1008976. doi: 10.1371/journal.pcbi.1008976 (PMC8121311; doi:10.1371/journal.pcbi.1008976)

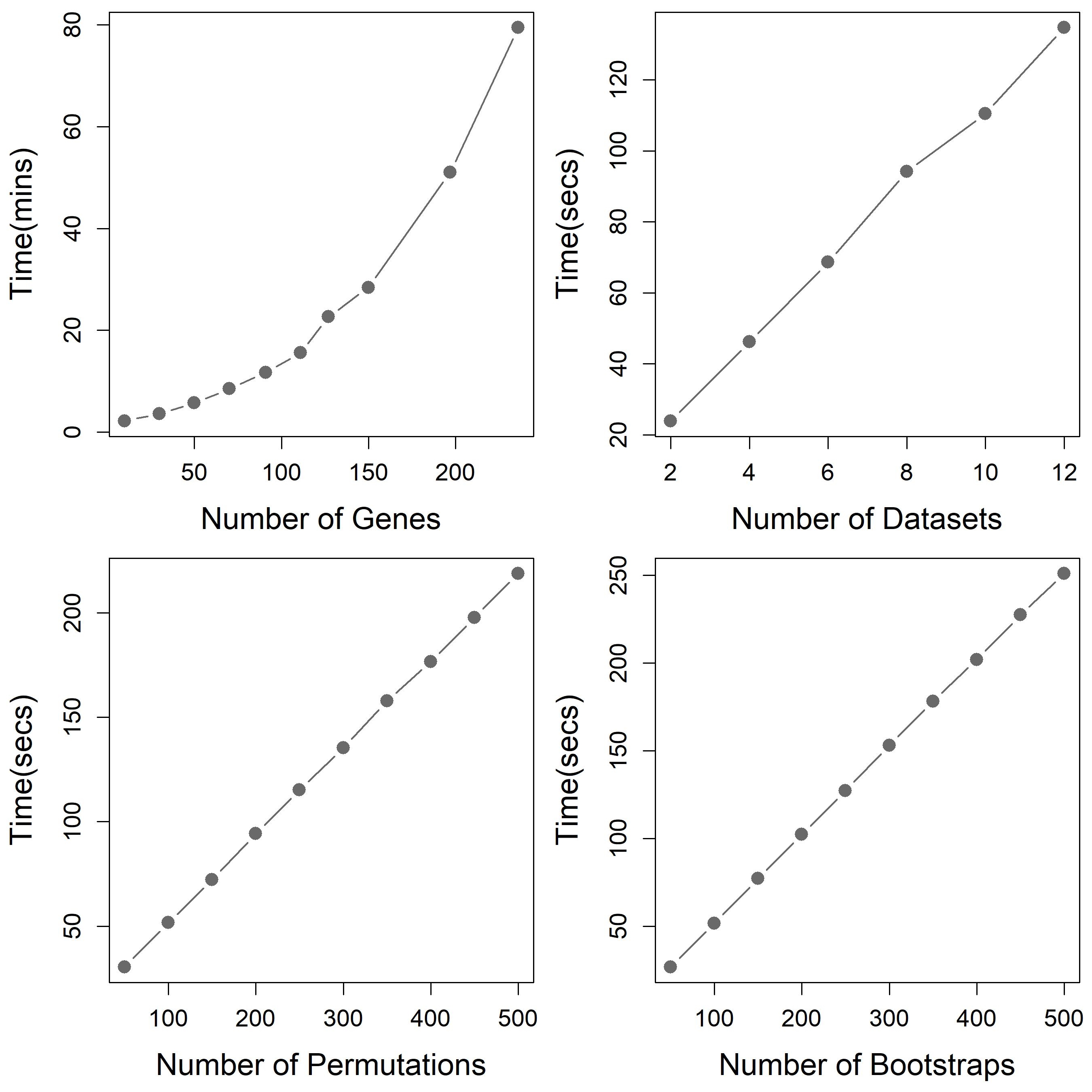

Supplement: S1 Fig — (TIFF) [file pcbi.1008976.s001.tiff]
