# Supplementary material for: MetaGSCA: A tool for meta-analysis of gene set differential coexpression
Source: PLoS Comput Biol. 2021 May 4;17(5):e1008976. doi: 10.1371/journal.pcbi.1008976 (PMC8121311; doi:10.1371/journal.pcbi.1008976)
Supplement: S1 Table — (DOCX) [file pcbi.1008976.s002.docx]

| **Pathway** | **Source** | **Size** |
| --- | --- | --- |
| Aurora C signaling | pid | 5 |
| Class IB PI3K non-lipid kinase events | pid | 5 |
| Gamma-aminobutyric acid synthesis | panther | 5 |
| Tetrahydrofolate biosynthesis | panther | 5 |
| Adenine and hypoxanthine salvage pathway | panther | 6 |
| Alternative NF-kappaB pathway | pid | 6 |
| Arginine biosynthesis | panther | 6 |
| Coenzyme A biosynthesis | panther | 6 |
| EphrinB-EPHB pathway | pid | 6 |
| Formyltetrahydroformate biosynthesis | panther | 6 |
| IFN gamma signaling(JAK1 JAK2 STAT1) | inoh | 6 |
| IL-10 signaling(JAK1 TYK2 STAT3) | inoh | 6 |
| IL-6 signaling(JAK1 JAK2 STAT3) | inoh | 6 |
| Mannose metabolism | panther | 6 |
| mRNA splicing | panther | 6 |
| N-acetylglucosamine metabolism | panther | 6 |
| P53 pathway feedback loops 1 | panther | 6 |
| PDGF receptor signaling network | pid | 6 |
| PLK3 signaling events | pid | 6 |
| 5-arachidonylglycerol_biosynthesis | panther | 7 |
| BMP signaling in Drosophila | inoh | 7 |
| FGF signaling pathway (D. melanogaster) | inoh | 7 |
| IL-12 signaling(JAK2 TYK2 STAT4) | inoh | 7 |
| IL-2 signaling(JAK1 JAK3 STAT5) | inoh | 7 |
| IL-3 signaling(JAK1 JAK2 STAT5) | inoh | 7 |
| IL-4 signaling(JAK1 JAK3 STAT6) | inoh | 7 |
| Porphyrin metabolism | inoh | 7 |
| a4b7 Integrin signaling | pid | 8 |
| EphrinA-EPHA pathway | pid | 8 |
| Pentose phosphate pathway | panther | 8 |
| S1P5 pathway | pid | 8 |
| Vitamin D metabolism and pathway | panther | 8 |
| Androgen;estrogene;progesterone biosynthesis | Merged from multiple pathways | 9 |
| Circadian clock system | panther | 9 |
| Effects of Botulinum toxin | pid | 9 |
| EGFR-dependent Endothelin signaling events | pid | 9 |
| Fructose galactose metabolism | panther | 9 |
| Glypican 3 network | pid | 9 |
| Growth hormone signaling(JAK2 STAT5) | inoh | 9 |
| Nicotine_degradation | panther | 9 |
| De novo pyrimidine ribonucleotides biosythesis | panther | 10 |
| Galactose metabolism | inoh | 10 |
| IL-13 signaling(JAK1 TYK2 STAT6) | inoh | 10 |
| LIF signaling(JAK1 JAK2 STAT3) | inoh | 10 |
| Pyrimidine Metabolism | panther | 10 |
| Rapid glucocorticoid signaling | pid | 10 |
| Salvage pyrimidine ribonucleotides | panther | 10 |
| TCA cycle | panther | 10 |
| VEGF and VEGFR signaling network | pid | 10 |
| ALK2 signaling events | pid | 11 |
| Alpha6 beta4 integrin-ligand interactions | pid | 11 |
| Heme biosynthesis | panther | 11 |
| IL-5 signaling(JAK1 JAK2 STAT1 STAT5) | inoh | 11 |
| Signaling mediated by p38-gamma and p38-delta | pid | 11 |
| Vasopressin synthesis | panther | 11 |
| Cholesterol biosynthesis | panther | 12 |
| De novo pyrimidine deoxyribonucleotide biosynthesis | panther | 12 |
| 5HT3 type receptor mediated signaling pathway | panther | 13 |
| DNA-PK pathway in nonhomologous end joining | pid | 13 |
| Phenylalanine degradation | inoh | 13 |
| Atypical NF-kappaB pathway | pid | 14 |
| Cell cycle | panther | 14 |
| IL5-mediated signaling events | pid | 14 |
| JNK signaling in the CD4+ TCR pathway | pid | 14 |
| Ras signaling in the CD4+ TCR pathway | pid | 14 |
| S1P4 pathway | pid | 14 |
| Arf6 downstream pathway | pid | 15 |
| BMP2 signaling(through TAK1) | inoh | 15 |
| ErbB receptor signaling network | pid | 15 |
| General transcription by RNA polymerase I | panther | 15 |
| IL-1 signaling pathway (through NF-kappaB) | inoh | 15 |
| JAK;STAT signaling pathway | Merged from multiple pathways | 15 |
| PAR4-mediated thrombin signaling events | pid | 15 |
| Sumoylation by RanBP2 regulates transcriptional repression | pid | 15 |
| Validated nuclear estrogen receptor beta network | pid | 15 |
| Aminosugars metabolism | inoh | 16 |
| LPA4-mediated signaling events | pid | 16 |
| Plasminogen activating cascade | panther | 16 |
| Steroids metabolism | inoh | 16 |
| Beta5 beta6 beta7 and beta8 integrin cell surface interactions | pid | 17 |
| Butanoate metabolism | inoh | 17 |
| Circadian rhythm pathway | pid | 17 |
| Glycolysis | panther | 17 |
| 5-Hydroxytryptamine degredation | panther | 18 |
| Axon guidance mediated by Slit;Robo | Merged from multiple pathways | 18 |
| Degradation of beta catenin | pid | 18 |
| DNA replication | panther | 18 |
| Axon guidance mediated by semaphorins | panther | 19 |
| EPHA2 forward signaling | pid | 19 |
| Glutamic acid and Glutamine metabolism | inoh | 19 |
| Hypoxia response via HIF activation | panther | 19 |
| Hypoxic and oxygen homeostasis regulation of HIF-1-alpha | pid | 19 |
| Regulation of cytoplasmic and nuclear SMAD2;3 signaling | Merged from multiple pathways | 20 |
| S1P1 pathway | pid | 20 |
| TGF-beta signaling(through TAK1) | inoh | 20 |
| Canonical Wnt signaling pathway | pid | 21 |
| E-cadherin signaling in keratinocytes | pid | 21 |
| IFN alpha signaling(JAK1 TYK2 STAT1 STAT2 STAT3) | inoh | 21 |
| Lysine degradation | inoh | 21 |
| NGF signaling pathway | inoh | 21 |
| Notch signaling pathway Diagram | inoh | 21 |
| p38 signaling mediated by MAPKAP kinases | pid | 21 |
| p53 pathway by glucose deprivation | panther | 21 |
| Sphingosine 1-phosphate (S1P) pathway | pid | 21 |
| Synaptic_vesicle_trafficking | panther | 21 |
| Alanine,Aspartic acid and Asparagine metabolism | inoh | 22 |
| Nicotinate and Nicotinamide metabolism | inoh | 22 |
| PDGFR-alpha signaling pathway | pid | 22 |
| Propanoate metabolism | inoh | 22 |
| Prostaglandin and Leukotriene metabolism | inoh | 22 |
| Alpha adrenergic receptor signaling pathway | panther | 23 |
| Histidine degradation | inoh | 23 |
| Insulin;IGF pathway-protein kinase B signaling cascade | Merged from multiple pathways | 23 |
| Methionine and Cysteine metabolism | inoh | 23 |
| Pentose phosphate cycle | inoh | 23 |
| Signaling events mediated by PRL | pid | 23 |
| Syndecan-3-mediated signaling events | pid | 23 |
| Canonical NF-kappaB pathway | pid | 24 |
| Endogenous_cannabinoid_signaling | panther | 24 |
| Fas(lowercase) signaling pathway | inoh | 24 |
| Metabotropic glutamate receptor group I pathway | panther | 24 |
| Plexin-D1 Signaling | pid | 24 |
| Signaling events mediated by the Hedgehog family | pid | 24 |
| Visual signal transduction; Cones | Merged from multiple pathways | 24 |
| Visual signal transduction; Rods | Merged from multiple pathways | 24 |
| Alpha9 beta1 integrin signaling events | pid | 25 |
| Cellular roles of Anthrax toxin | pid | 25 |
| C-MYC pathway | pid | 25 |
| S1P3 pathway | pid | 25 |
| Toll-like receptor signaling pathway (through JNK cascade) | inoh | 25 |
| TRAIL signaling pathway | pid | 25 |
| ALK1 signaling events | pid | 26 |
| De novo purine biosynthesis | panther | 26 |
| Endogenous TLR signaling | pid | 26 |
| Ephrin B reverse signaling | pid | 26 |
| Glypican 1 network | pid | 26 |
| IL27-mediated signaling events | pid | 26 |
| Integrin family cell surface interactions | pid | 26 |
| RXR and RAR heterodimerization with other nuclear receptor | pid | 26 |
| S1P2 pathway | pid | 26 |
| Toll-like receptor signaling pathway (p38 cascade) | inoh | 26 |
| Toll-like receptor signaling pathway (through ECSIT, MEKK1, MKKs, JNK cascade) | inoh | 26 |
| Toll-like receptor signaling pathway (through ECSIT, MEKK1, MKKs, p38 cascade) | inoh | 26 |
| VEGFR3 signaling in lymphatic endothelium | pid | 26 |
| Adrenaline and noradrenaline biosynthesis | panther | 27 |
| Citrate cycle | inoh | 27 |
| Folate metabolism | inoh | 27 |
| IL3-mediated signaling events | pid | 27 |
| Interferon-gamma signaling pathway | panther | 27 |
| Arf1 pathway | pid | 28 |
| EGF signaling pathway Diagram | inoh | 28 |
| Fructose and Mannose metabolism | inoh | 28 |
| GPCR GroupI metabotropic glutamate receptor signaling pathway | inoh | 28 |
| Nephrin;Neph1 signaling in the kidney podocyte | Merged from multiple pathways | 28 |
| Nicotine pharmacodynamics pathway | panther | 28 |
| Reelin signaling pathway | pid | 28 |
| BARD1 signaling events | pid | 29 |
| Beta2 integrin cell surface interactions | pid | 29 |
| Calcium signaling in the CD4+ TCR pathway | pid | 29 |
| IL-1 signaling pathway (through p38 cascade) | inoh | 29 |
| Insulin;IGF pathway-mitogen activated protein kinase kinase;MAP kinase cascade | Merged from multiple pathways | 29 |
| p38 MAPK signaling pathway | pid | 29 |
| VEGFR1 specific signals | pid | 29 |
| Wnt signaling network | pid | 29 |
| Axon guidance mediated by netrin | panther | 30 |
| General transcription regulation | panther | 30 |
| IGF1 pathway | pid | 30 |
| IL2 signaling events mediated by STAT5 | pid | 30 |
| Insulin-mediated glucose transport | pid | 30 |
| Nectin adhesion pathway | pid | 30 |
| Retinoic acid receptors-mediated signaling | pid | 30 |
| Valine,Leucine and Isoleucine degradation | inoh | 30 |
| IL-1 signaling pathway (through JNK cascade) | inoh | 31 |
| Lissencephaly gene (LIS1) in neuronal migration and development | pid | 31 |
| Nongenotropic Androgen signaling | pid | 31 |
| Regulation of CDC42 activity | pid | 31 |
| Regulation of p38-alpha and p38-beta | pid | 31 |
| Toll-like receptor signaling pathway (trough NF-kappaB) | inoh | 31 |
| Tyrosine metabolism | inoh | 31 |
| amb2 Integrin signaling | pid | 32 |
| Beta1 adrenergic receptor signaling pathway | panther | 32 |
| Netrin-mediated signaling events | pid | 32 |
| Osteopontin-mediated events | pid | 32 |
| p38 MAPK pathway | panther | 32 |
| Syndecan-4-mediated signaling events | pid | 32 |
| TNFR1 signaling pathway | inoh | 32 |
| Aurora A signaling | pid | 33 |
| Enkephalin release | panther | 33 |
| EPHA forward signaling | pid | 33 |
| EPO signaling pathway | pid | 33 |
| Hedgehog signaling pathway | inoh,panther | 33 |
| HIV-1 Nef; Negative effector of Fas and TNF-alpha | Merged from multiple pathways | 33 |
| IL12 signaling mediated by STAT4 | pid | 33 |
| Noncanonical Wnt signaling pathway | pid | 33 |
| Pyruvate metabolism | inoh, panther | 33 |
| Alpha4 beta1 integrin signaling events | pid | 34 |
| Alpha-synuclein signaling | pid | 34 |
| ATM pathway | pid | 34 |
| HIF-2-alpha transcription factor network | pid | 34 |
| IL1-mediated signaling events | pid | 34 |
| Regulation of Ras family activation | pid | 34 |
| Trk receptor signaling mediated by the MAPK pathway | pid | 34 |
| Angiotensin_II-stimulated_signaling_through_G_proteins_and_beta-arrestin | panther | 35 |
| Class I PI3K signaling events mediated by Akt | pid | 35 |
| ErbB4 signaling events | pid | 35 |
| GABA-B_receptor_II_signaling | panther | 35 |
| Heterotrimeric G-protein signaling pathway-rod outer segment phototransduction | panther | 35 |
| HGF signaling pathway | inoh | 35 |
| IL8- and CXCR2-mediated signaling events | pid | 35 |
| Signaling mediated by p38-alpha and p38-beta | pid | 35 |
| Arf6 signaling events | pid | 36 |
| CD40;CD40L signaling | Merged from multiple pathways | 36 |
| FAS (CD95) signaling pathway | pid | 36 |
| GPCR Adenosine A2A receptor signaling pathway | inoh | 36 |
| IGF1 signaling pathway | inoh | 36 |
| N-cadherin signaling events | pid | 36 |
| Trk receptor signaling mediated by PI3K and PLC-gamma | pid | 36 |
| E-cadherin signaling in the nascent adherens junction | pid | 37 |
| FAS(uppercase) signaling pathway | panther | 37 |
| IL23-mediated signaling events | pid | 37 |
| Validated transcriptional targets of AP1 family members Fra1 and Fra2 | pid | 37 |
| Blood coagulation | panther | 38 |
| CXCR3-mediated signaling events | pid | 38 |
| Glycine and Serine metabolism | inoh | 38 |
| GMCSF-mediated signaling events | pid | 38 |
| IL2 signaling events mediated by PI3K | pid | 38 |
| Internalization of ErbB1 | pid | 38 |
| Syndecan-2-mediated signaling events | pid | 38 |
| ATR signaling pathway | pid | 39 |
| Regulation of RAC1 activity | pid | 39 |
| Signaling events regulated by Ret tyrosine kinase | pid | 39 |
| EPHB forward signaling | pid | 40 |
| Signaling events mediated by TCPTP | pid | 40 |
| Aurora B signaling | pid | 41 |
| ErbB2;ErbB3 signaling events | Merged from multiple pathways | 41 |
| Glycolysis and Gluconeogenesis | inoh | 41 |
| Signaling events mediated by HDAC Class III | pid | 41 |
| 5HT1 type receptor mediated signaling pathway | panther | 42 |
| BMP receptor signaling | pid | 42 |
| FOXM1 transcription factor network | pid | 42 |
| Inositol phosphate metabolism | inoh | 42 |
| Ionotropic glutamate receptor pathway | panther | 42 |
| Metabotropic glutamate receptor group II pathway | panther | 42 |
| Plasma membrane estrogen receptor signaling | pid | 42 |
| Syndecan-1-mediated signaling events | pid | 42 |
| Urokinase-type plasminogen activator (uPA) and uPAR-mediated signaling | pid | 42 |
| IFN-gamma pathway | pid | 43 |
| LKB1 signaling events | pid | 43 |
| p53 pathway feedback loops 2 | panther | 43 |
| PAR1-mediated thrombin signaling events | pid | 43 |
| PI3 kinase pathway | panther | 43 |
| Stabilization and expansion of the E-cadherin adherens junction | pid | 43 |
| Beta3 integrin cell surface interactions | pid | 44 |
| Toll receptor signaling pathway | panther | 44 |
| Ubiquitin proteasome pathway | panther | 44 |
| CD4 T cell receptor signaling (ERK cascade) | inoh | 45 |
| FOXA1 transcription factor network | pid | 45 |
| FOXA2 and FOXA3 transcription factor networks | pid | 45 |
| Oxidative stress response | panther | 45 |
| 5HT2 type receptor mediated signaling pathway | panther | 46 |
| a6b1 and a6b4 Integrin signaling | pid | 46 |
| Class I PI3K signaling events | pid | 46 |
| Integrin-linked kinase signaling | pid | 46 |
| Presenilin action in Notch and Wnt signaling | pid | 46 |
| RhoA signaling pathway | pid | 46 |
| TNF receptor signaling pathway | pid | 46 |
| Insulin Pathway | pid | 47 |
| Transcription regulation by bZIP transcription factor | panther | 47 |
| Validated transcriptional targets of deltaNp63 isoforms | pid | 47 |
| Arf6 trafficking events | pid | 48 |
| Arginine and Proline metabolism | inoh | 48 |
| Fanconi anemia pathway | pid | 48 |
| IL6-mediated signaling events | pid | 48 |
| Notch-mediated HES;HEY network | Merged from multiple pathways | 48 |
| PLK1 signaling events | pid | 48 |
| Pyrimidine Nucleotides and Nucleosides metabolism | inoh | 48 |
| Regulation of RhoA activity | pid | 48 |
| Ceramide signaling pathway | pid | 49 |
| Hedgehog signaling events mediated by Gli proteins | pid | 49 |
| Angiopoietin receptor Tie2-mediated signaling | pid | 50 |
| Calcineurin-regulated NFAT-dependent transcription in lymphocytes | pid | 50 |
| FoxO family signaling | pid | 50 |
| GPCR Dopamine D1like receptor signaling pathway | inoh | 50 |
| Posttranslational regulation of adherens junction stability and dissassembly | pid | 50 |
| PNAT | panther | 51 |
| CD4 T cell receptor signaling (JNK cascade) | inoh | 53 |
| Muscarinic acetylcholine receptor 2 and 4 signaling pathway | panther | 53 |
| Signaling events mediated by PTP1B | pid | 53 |
| Signaling events mediated by Stem cell factor receptor (c-Kit) | pid | 53 |
| TGF-beta receptor signaling | pid | 53 |
| IL2-mediated signaling events | pid | 54 |
| Muscarinic acetylcholine receptor 1 and 3 signaling pathway | panther | 54 |
| RAC1 signaling pathway | pid | 54 |
| Regulation of Androgen receptor activity | pid | 54 |
| Role of Calcineurin-dependent NFAT signaling in lymphocytes | pid | 54 |
| Validated transcriptional targets of TAp63 isoforms | pid | 54 |
| B cell activation | panther | 56 |
| TGF-beta_BMP Diagram(MolecularVariation) | inoh | 56 |
| Thromboxane A2 receptor signaling | pid | 56 |
| Thyrotropin-releasing hormone receptor signaling pathway | panther | 56 |
| Caspase Cascade in Apoptosis | pid | 57 |
| CD4 T cell receptor signaling (NF-kB cascade) | inoh | 57 |
| Notch signaling pathway | panther, pid | 58 |
| Signaling events mediated by HDAC Class II | pid | 58 |
| ATF-2 transcription factor network | pid | 59 |
| p53 pathway | panther, pid | 59 |
| FGF signaling pathway | inoh, panther, pid | 60 |
| SHP2 signaling | pid | 60 |
| Coregulation of Androgen receptor activity | pid | 61 |
| IL12-mediated signaling events | pid | 62 |
| Alzheimer disease-amyloid secretase pathway | panther | 63 |
| Neurotrophic factor-mediated Trk receptor signaling | pid | 63 |
| Signaling events mediated by focal adhesion kinase | pid | 63 |
| Validated targets of C-MYC transcriptional repression | pid | 63 |
| Endothelins | pid | 64 |
| Fc-epsilon receptor I signaling in mast cells | pid | 64 |
| T cell activation | panther | 64 |
| Tryptophan degradation | inoh | 64 |
| LPA receptor mediated events | pid | 65 |
| Metabotropic glutamate receptor group III pathway | panther | 65 |
| Validated nuclear estrogen receptor alpha network | pid | 65 |
| IL4-mediated signaling events | pid | 66 |
| Integrins in angiogenesis | pid | 66 |
| mTOR signaling pathway | pid | 66 |
| HIF-1-alpha transcription factor network | pid | 67 |
| Regulation of retinoblastoma protein | pid | 67 |
| Downstream signaling in na??ve CD8+ T cells | pid | 69 |
| Signaling events mediated by VEGFR1 and VEGFR2 | pid | 69 |
| TCR signaling in na??ve CD4+ T cells | pid | 69 |
| BCR signaling pathway | pid | 70 |
| Beta1 integrin cell surface interactions | pid | 70 |
| Cytoskeletal regulation by Rho GTPase | panther | 70 |
| Ras Pathway | panther | 70 |
| Regulation of Telomerase | pid | 70 |
| AP-1 transcription factor network | pid | 71 |
| CDC42 signaling events | pid | 71 |
| p75(NTR)-mediated signaling | pid | 74 |
| E2F transcription factor network | pid | 77 |
| Interleukin signaling pathway | panther | 78 |
| Insulin receptor signaling (C. elegans) | inoh | 79 |
| p73 transcription factor network | pid | 80 |
| Regulation of nuclear beta catenin signaling and target gene transcription | pid | 80 |
| Signaling events mediated by Hepatocyte Growth Factor Receptor (c-Met) | pid | 80 |
| Validated targets of C-MYC transcriptional activation | pid | 80 |
| TGF-beta_super_family_signaling_pathway(canonical) | inoh | 81 |
| Insulin receptor signaling (Mammal) | inoh | 82 |
| Regulation of nuclear SMAD2;3 signaling | Merged from multiple pathways | 82 |
| Glucocorticoid receptor regulatory network | pid | 85 |
| Nicotinic acetylcholine receptor signaling pathway | panther | 86 |
| C-MYB transcription factor network | pid | 87 |
| CXCR4-mediated signaling events | pid | 87 |
| CD4 T cell receptor signaling | inoh | 88 |
| Parkinson disease | panther | 89 |
| Signaling events mediated by HDAC Class I | pid | 89 |
| Canonical Wnt signaling pathway Diagram | inoh | 91 |
| C. elegans endoderm induction Wnt signaling pathway Diagram | inoh | 91 |
| Drosophila Wingless;Wnt signaling pathway Diagram | Merged from multiple pathways | 91 |
| Mammalian Wnt signaling pathway Diagram | inoh | 91 |
| TGF-beta signaling pathway | panther | 91 |
| Xenopus axis formation Wnt signaling pathway Diagram | inoh | 91 |
| B cell receptor signaling | inoh | 92 |
| Purine nucleotides and Nucleosides metabolism | inoh | 104 |
| ErbB1 downstream signaling | pid | 105 |
| Apoptosis signaling pathway | panther | 108 |
| Alzheimer disease-presenilin pathway | panther | 111 |
| EGF receptor signaling pathway | panther | 115 |
| Heterotrimeric G-protein signaling pathway-Gq alpha and Go alpha mediated pathway | panther | 118 |
| Huntington disease | panther | 118 |
| PDGFR-beta signaling pathway | pid | 125 |
| PDGF signaling pathway | inoh, panther | 125 |
| Integrin signaling pathway | inoh | 127 |
| Direct p53 effectors | pid | 140 |
| Gastrin_CCK2R_240212 | panther | 149 |
| Cadherin signaling pathway | panther | 150 |
| Angiogenesis | panther | 152 |
| Heterotrimeric G-protein signaling pathway-Gi alpha and Gs alpha mediated pathway | panther | 152 |
| Integrin signalling pathway | panther | 166 |
| Inflammation mediated by chemokine and cytokine signaling pathway | panther | 197 |
| Drosophila Toll-like receptor signaling | inoh | 236 |
