# Supplementary material for: MetaGSCA: A tool for meta-analysis of gene set differential coexpression
Source: PLoS Comput Biol. 2021 May 4;17(5):e1008976. doi: 10.1371/journal.pcbi.1008976 (PMC8121311; doi:10.1371/journal.pcbi.1008976)
Supplement: S2 Table — (DOCX) [file pcbi.1008976.s003.docx]

| **Dataset** | **Study theme (Condition 1 vs 2)** | **Dataset name** | **Number of Samples with Condition 1** | **Number of Samples with Condition 2** |
| --- | --- | --- | --- | --- |
| CKD | Chronic kidney disease (disease vs. normal) | CKD-own | 17 | 16 |
|  |  | GSE37171 | 21 | 20 |
|  |  | GSE62792 | 12 | 6 |
| NSCLC | Non-small cell lung cancer (lung squamous cell carcinoma vs. lung adenocarcinoma) | GSE10245 | 40 | 18 |
|  |  | GSE11969 | 90 | 21 |
|  |  | GSE41271 | 176 | 78 |
|  |  | GSE42127 | 131 | 43 |
| TCGA unpaired* | Breast invasive carcinoma | BRCA | 1102 | 113 |
|  | Colon adenocarcinoma | COAD | 451 | 41 |
|  | Head and neck squamous cell carcinoma | HNSC | 522 | 44 |
|  | Kidney renal clear cell carcinoma | KIRC | 534 | 72 |
|  | Kidney renal papillary cell carcinoma | KIRP | 291 | 32 |
|  | Liver hepatocellular carcinoma | LIHC | 374 | 50 |
|  | Lung adenocarcinoma | LUAD | 517 | 59 |
|  | Lung squamous cell carcinoma | LUSC | 502 | 51 |
|  | Prostate adenocarcinoma | PRAD | 498 | 52 |
|  | Stomach adenocarcinoma | STAD | 415 | 35 |
|  | Thyroid carcinoma | THCA | 513 | 59 |

*Shown in the table are sample sizes for the unpaired TCGA analysis. The paired analysis used two groups of identical size, same as indicated in the last column (number of samples with Condition 2) for the TCGA unpaired analysis.
